# Patient safety and the second victim phenomenon in nursing and medical curricula: A qualitative study

**DOI:** 10.1016/j.ijnsa.2025.100403

**Published:** 2025-08-06

**Authors:** Einav Srulovici, Mary-Elizabeth Tumelty, Ivana Meszaros Skoumalova, Patricia Vella Bonanno, Erika Kubilienė, José Joaquín Mira, Valentina Marinkovic, Anat Rafaeli, Reinhard Strametz, Susanna Tella, Anu Venesoja, Žymantė Jankauskienė, Sandra C. Buttigieg

**Affiliations:** a The Cheryl Spencer Department of Nursing, University of Haifa, Israel; b School of Law, University College Cork, Ireland; c Department of Health Psychology and Research Methodology, Faculty of Medicine, P. J. Safarik University, Slovakia; d Department of Health Systems Management and Leadership, Faculty of Health Sciences, University of Malta, Malta; e Faculty of Health Care, Vilniaus kolegija / Higher Education Institution, Lithuania; f Health Psychology Department, Universidad Miguel Hernández, Sant Joan, Spain; g Department of Social Pharmacy and Pharmaceutical Legislation, Faculty of Pharmacy, University of Belgrade, Belgrade, Serbia; h Technion - Israel Institute of Technology, Israel; i Wiesbaden Institute for Healthcare Economics and Patient Safety (WiHelP), RheinMain UAS, Wiesbaden/Germany; j Faculty of Health and Social Care, LAB University of Applied Sciences, Lappeenranta, Finland; k Department of Emergency Care and Services, University of Helsinki and Helsinki University Hospital, Helsinki, Finland

**Keywords:** Patient safety (MeSH), Safety management (MeSH), Curriculum (MeSH), Education, nursing (MeSH), Education, medical (MeSH)

## Abstract

**Background:**

The second victim phenomenon—emotional and psychological distress experienced by healthcare professionals following adverse events—is increasingly recognized. However, its integration into formal nursing and medical curricula remains limited across Europe, despite its relevance to patient safety, as well as student and clinician well-being.

**Objectives:**

To explore how patient safety and second victim content are incorporated into undergraduate and postgraduate nursing and medical curricula and to identify the barriers and facilitators influencing such integration across Europe.

**Design:**

A qualitative cross-national interview study.

**Settings:**

Medical and nursing education institutions in 10 European countries representing northern, southern, central, and eastern regions.

**Participants:**

Nineteen healthcare education leaders (department heads or senior faculty) from nursing and medical programs were selected purposefully based on their leadership roles and curricular oversight responsibilities. Inclusion criteria required at least 5 years of experience in curriculum development at both undergraduate and postgraduate levels; there were no dropouts.

**Methods:**

Semi-structured interviews were conducted in participants' native languages, translated to English, and analyzed using thematic analysis. Themes were developed inductively by a multidisciplinary research team across countries.

**Results:**

Five major themes were identified: (1) Recognition of patient safety and the second victim phenomenon; (2) Curriculum development and implementation; (3) Training content and delivery; (4) Student and educator engagement; and (5) Continuous professional development. Although awareness of the second victim concept was high, formal curricular integration was rare. Barriers included curriculum overload, regulatory rigidity, and limited faculty preparedness. Facilitators included interdisciplinary collaboration, student advocacy, and openness to innovative pedagogies.

**Conclusions:**

Despite broad recognition of the second victim phenomenon, its integration into European healthcare curricula remains minimal. Strategic curriculum reforms supported by interdisciplinary collaboration, institutional leadership, and student engagement may be essential to bridge the gap between awareness and educational practice. We have offered actionable guidance for advancing patient safety and clinician resilience through formal education.

**What is already known about the topic**

• The second victim phenomenon, describing the emotional impact of adverse events on healthcare providers, is increasingly recognized across healthcare systems but remains largely absent from healthcare curricula.
• Patient safety education is hindered by curriculum overload, faculty unfamiliarity, and regulatory constraints, despite being essential to professional development.
• Researchers have emphasized the importance of addressing emotional resilience and coping with clinical errors in health education, but such competencies are frequently underprioritized compared to technical knowledge.

**What this paper adds**

• We identified significant gaps in curricular integration of second victim education, despite high awareness among European healthcare education leaders.
• We have revealed structural barriers that hinder the incorporation of second victim education within formal healthcare training programs.
• We have provided empirically-grounded insights to inform stakeholder-driven curriculum reforms aimed at enhancing resilience and patient safety in healthcare education.

## 1 Introduction

Within the intricate landscape of healthcare, patient safety incidents present multifaceted challenges, impacting patient well-being, healthcare providers, and the broader healthcare system (Classen et al., 2011; Busch et al., 2020). This complexity underscores the critical need for a comprehensive approach to patient safety to minimize harm to patients and secondary harms to healthcare professionals (Panagioti et al., 2019).

Central to this discourse is the phenomenon known as the second victim, a term originally coined by Wu (2000), which describes the emotional and psychological distress experienced by healthcare professionals following patient safety incidents. Scott et al. (2009) further elaborated on this concept, defining second victims as healthcare providers involved in adverse patient events, medical errors, or patient-related injuries, who subsequently experience trauma due to the incident. More recently, Vanhaecht et al. (2022) expanded on this definition, emphasizing that any healthcare worker, whether directly or indirectly involved in an unexpected adverse event or error, can be affected, with potential negative consequences for both the individual and the organization.

Evidence shows the widespread prevalence of second victim syndrome among healthcare professionals across various countries and specialties. Researchers have shown consistently that a significant proportion of healthcare workers are affected, with rates ranging from 56 % to 89 %, as seen in examples from Germany (Strametz et al., 2021a, 2021b), Austria (Potura et al., 2023), and Spain (Mira et al., 2015). Similarly, investigators conducting a recent systematic review found that 58 % of intensive care unit healthcare professionals experience second victim syndrome (Naya et al., 2023). The distress associated with second victim syndrome can manifest in such symptoms as difficult memories, anxiety, anger, regret, stress, fear of future mistakes, shame, guilt, and difficulty sleeping (Busch et al., 2020). These findings underscore the global scope of the issue and the critical importance of providing support to healthcare professionals’ post-incident, not only for their well-being but also for ensuring patient safety (Mira et al., 2024a; Wu et al., 2020).

Notably, the second victim phenomenon is not confined to a specific stage of a healthcare professional's career but affects individuals across the spectrum of experience. From nursing students, who are the future of the healthcare workforce, to early-career practitioners and seasoned professionals, all can find themselves entangled in patient incidents and struggling with traumatic symptoms in the aftermath (Van Slambrouck et al., 2021; Mira et al., 2024b). Krogh et al. (2023) further demonstrated that students in health-related programs who merely witness serious adverse events can exhibit psychological distress similar to that of qualified professionals, despite having only observer status. This early vulnerability highlights the need for preventive and supportive interventions throughout their educational program, including both academic and clinical learning environments. As the literature underscores the pressing need to address the consequences of the second victim phenomenon, a question emerges concerning the preparedness of educational institutions in the health sciences to equip students and early-career practitioners alike (Slambrouck et al., 2021).

With this in mind, the need for a comprehensive patient safety curriculum arises as a beacon of proactive intervention (World Health Organization [WHO], 2011). The proposition is clear: early training in patient safety coupled with targeted support mechanisms provides a holistic framework for healthcare professionals and students with the potential to mitigate the impact of becoming secondary victims (Mira et al., 2024b; Rodriguez, Scott, 2018; Tavares et al., 2022). Beyond individual well-being, such interventions serve as catalysts for fortifying the foundations of patient safety. For example, one group of researchers showed that, while patient safety is addressed in nursing and medical schools, it is insufficient, and the focus on the second victim phenomenon remains scant (Sánchez-García et al., 2023). This gap may be due to curriculum overload, a lack of awareness among educators, limited resources, cultural reluctance to address errors, and a predominant focus on technical skills over emotional competencies. In the global action plan for patient safety published by the World Health Organization, it is emphasized that patient safety should be a central component in all healthcare education (WHO, 2011, 2021). However, some barriers have been identified in implementation. For example, teachers are often unfamiliar with teaching patient safety and associated topics, and academic institutions are hesitant to introduce new content into an already-crowded curriculum (Gil-Hernández et al., 2024; Vanhaecht et al., 2022).

In this paper, we have explored the complex terrain of healthcare education, with a specific focus on undergraduate and postgraduate health professional (nursing and medicine) curricula. By examining the facilitators and barriers to implementing standalone training courses on patient safety and the second victim phenomenon, we have provided associated insights. Acknowledging the dynamic nature of healthcare education and its entwinement with cultural and regional contexts, we sought to contribute to the ongoing dialogue on bolstering patient safety education and fostering resilience among healthcare providers across diverse educational settings (Carrillo et al., 2022). Therefore, we aimed to identify common facilitators and barriers influencing curricular changes regarding patient safety and the second victim phenomenon across Europe.

## 2 Methods

### 2.1 Study design

This was a multinational qualitative study under the auspices of the European Researchers' Network Working on Second Victims consortium. The consortium aims to explore and address the second victim phenomenon in healthcare across Europe. The study protocol provided a comprehensive guide for the interviews, ensuring consistency across different countries. Semi-structured interviews were conducted from April 2023 to June 2023.

### 2.2 Study setting, sampling, and participants

The sample consisted of leaders of nursing and medical programs, referred to as “healthcare education leaders” throughout this paper. Participants were from 10 European countries: Finland, Iceland, Ireland, Israel, Lithuania, Norway, Malta, Serbia, Slovakia, and Spain. These countries were chosen to ensure representation from diverse geographical regions across Europe—north, south, central, and east—capturing a wide range of educational approaches and healthcare challenges and providing a comprehensive perspective on healthcare education leadership. A total of 19 healthcare education leaders were recruited via purposeful sampling, aiming to capture a wide range of perspectives. Inclusion criteria required participants to have a minimum of 5 years of experience in curriculum development at both undergraduate and postgraduate levels.

Participants were initially contacted via email by members of the national research teams. The invitation included a brief description of the study, its aims, and ethical assurances. Follow-up communication and scheduling of interviews were also conducted by email or phone, depending on participant preference. Each country had two participants, except for Ireland, which had only one participant due to logistical constraints. Approximately 85 % held senior academic positions, such as professors, department heads, or program directors, while over 70 % had prior or concurrent clinical roles, including hospital directors, internal medicine specialists, and advanced practice nurses. Most participants (around 90 %) had over 15 years of experience in higher education or healthcare leadership, and a significant portion (over 60 %) were directly involved in curriculum development, quality assurance, or national educational reforms—particularly in areas related to patient safety and healthcare quality. Thus, participants had extensive knowledge and experience in medical and nursing education at both undergraduate and postgraduate levels. This diverse sample provided a comprehensive trans-European overview of patient safety and the second victim phenomenon in healthcare education.

Data saturation was reached when no new themes or significant insights emerged from the interviews, confirming the adequacy of the sample for thematic analysis.

### 2.3 Procedure and measures

Semi-structured interviews were conducted with each participant, lasting between 20 and 50 min. All interviews were conducted via Zoom, enabling standardized yet flexible engagement across the 10 participating countries. The interviews were carried out in the language of each respective country and subsequently translated into English for analysis, recording, and synthesis, ensuring that no relevant nuances were lost in the translation process. The interview guide included questions such as: 1) “Tell me a bit about current patient safety courses/education in your school? What do they contain? Is there anything you feel is currently missing?” and 2) “Have you heard of the second victim phenomenon? Is that something that is currently addressed in health professionals’ education?”

The interviews aimed to elicit detailed insights into the current state of patient safety education and the inclusion of the second victim phenomenon in healthcare curricula.

### 2.4 Researcher reflexivity

The interviews were conducted by members of the European Researchers' Network Working on Second Victims consortium, a multidisciplinary team of 14 researchers (majority of females – 93 %) from 10 European countries, with expertise in nursing, medicine, psychology, law, health systems, and education. Their diverse cultural and disciplinary backgrounds enriched data collection and interpretation but also introduced varying assumptions. Most team members hold senior academic or clinical positions and are deeply engaged in patient safety and second victim research. The team recognized that their shared values—such as a commitment to improving clinician well-being—could influence data interpretation. To mitigate potential bias arising from these values, they employed reflexive strategies, including team discussions, assumption tracking, and cross-country peer debriefing. These reflexive efforts were further strengthened by the team’s geographical and cultural diversity, which introduced a range of perspectives and helped surface both common and context-specific insights. Together, this interplay between reflexivity and diversity supported a balanced and credible interpretation of the findings.

### 2.5 Trustworthiness and rigor

To ensure methodological rigor, we implemented several strategies aligned with established trustworthiness criteria (Lincoln and Guba, 1985). Credibility was enhanced through investigator triangulation, with multiple researchers independently analyzing data before collaborative theme development (Nowell et al., 2017), and member checking with selected participants.

Transferability was supported by providing rich contextual descriptions of participants and settings across ten European countries, enabling readers to assess applicability to their contexts (Connelly, 2016). Dependability was established through a systematic audit trail documenting methodological decisions and regular team meetings to ensure analytical consistency (Tobin and Begley, 2004). For confirmability, researchers practiced reflexivity by documenting potential biases, while our multinational, multidisciplinary team composition helped mitigate individual interpretive biases (Finlay, 2002).

### 2.6 Ethical considerations

Verbal informed consent was obtained from all participants at the beginning of each interview, and interviews were recorded with participants’ consent. We adhered to the guidelines of the Declaration of Helsinki (World Medical Association, 2013), and the recorded interviews were disposed of after transcription. Ethical approval was granted by the University of Haifa’s Research Ethics Committee (approval no 094/23). Participation was voluntary, and participants could withdraw from the study at any time without any consequences.

### 2.7 Analysis

Thematic analysis was employed to analyze the data, following the phases outlined by Braun and Clarke (2006). Three authors (MJH, MET, IS) independently familiarized themselves with the data, and then organized these codes into potential themes reflecting the results. During the analysis, five main themes were identified, each subdivided into categories based on whether they acted as facilitators or barriers to implementing changes in the curricula. The final themes were reviewed and named by the authors (MJH, HB, MET, IS), culminating in producing a comprehensive report.

## 3 Findings

### 3.1 Main themes

The five themes identified were: 1) Recognition and Understanding of Patient Safety and Second Victim Phenomenon, 2) Curriculum Development and Implementation, 3) Content and Delivery of Training, 4) Student and Educator Engagement and Impact, and 5) Continuous Professional Development and Future Directions. Each of the five themes could act as a facilitator or barrier to implementing patient safety and second victim stand-alone training courses in the undergraduate and postgraduate health professional curricula, depending on the perspective and context of the reply. Each theme began with a brief interpretive overview, followed by representative participant quotes and analysis to illustrate the theme’s key dimensions.

### 3.2 Theme 1: recognition and understanding of patient safety and the second victim phenomenon

This theme explored how participants recognized the significance of patient safety and the second victim phenomenon in healthcare education. While many were familiar with the concept, most noted its limited integration into formal curricula. The theme reflected the gap between widespread awareness and structured educational implementation. This theme supported the study’s aim by identifying common barriers to curricular change related to second victim education across European contexts.

#### 3.2.1 Recognition of the impact of adverse events on healthcare workers

Participants consistently recognized the profound impact of adverse events on both patients and healthcare workers. The concept of the second victim, while familiar to many, was seldom included in formal educational curricula. This gap underscored a significant area for improvement in healthcare education.

"Patients die, and health professionals get ruined" [i.e., experience emotional and psychological breakdown]. "Because they have hurt others. They have not worked with the consequences of that…" (Participant A, Norway)

“They [educators] should share previous experience about medical errors. I do that during the lecture, because I realise that is important to teach the student how to protect the patient but also themselves” (Participant A, Serbia)

“I was interrogated by the CID [critical incident debriefing] with three other colleagues separately—an element of second victim as well, even though I don't think this case led to consequences for any patients. But possibly there was an element of second victim when it happened 20 years ago.” (Participant B, Malta).

#### 3.2.2 Awareness and understanding of the second victim phenomenon

The participants stressed that understanding the second victim phenomenon was crucial not only for the well-being of healthcare workers but also for maintaining high standards of patient safety. They noted that medical errors were often systemic rather than individual failures, indicating a need for comprehensive educational approaches that addressed the broader context of patient safety. Despite this awareness, the incorporation of the second victim phenomenon into the curricula of healthcare professionals remained limited, suggesting a disconnect between recognized needs and educational practices. In several countries, participants were unfamiliar with the term “second victim,” even though they clearly recognized the emotional and professional impact of adverse events on clinicians. This suggested a gap not in experiential understanding but in the formal conceptual framing of the issue, which may limit its visibility within curricula and institutional discourse.

“These include the unfamiliarity of professors with teaching patient safety as a new area of knowledge and learning, the reluctance of academic institutions to teach knowledge outside clinical disciplines to health sciences students, as well as the failure of education to keep pace with technological and system advances in safe care.” (Participant B, Spain)

“I think this topic—the second victim phenomenon—is not currently in our curriculum. I have heard about it, but for example, I don’t include it in any of my teaching modules. I think it’s important, relevant, and advanced, and it should be included.” (Participant B, Lithuania)

"Yes, it is [familiar]. But we also mean that we do not do it to a certain extent, so we see that we need further development." (Participant A, Norway)

#### 3.2.3 Acknowledgement of the importance of patient safety education

This recognition acted as a facilitator for advocating changes in the curriculum but also highlighted barriers due to the current lack of structured educational content on the second victim phenomenon.

"…formal education talks about this, but in actual clinical practice, future health care professionals face a patient safety culture that is more bureaucratic or reactive only when an adverse event occurs." (Participant A, Lithuania)

"I think patient safety has been a common thread in our teaching for years, and I believe that it is quite strongly involved in teaching all the time. But that this second victim is probably one that we should probably take more control of, this is probably really something we should invest in.” (Participant A, Finland)

### 3.3 Theme 2: curriculum development and implementation

This theme focused on the structural and institutional challenges of integrating patient safety and second victim content into existing healthcare curricula. Participants highlighted persistent barriers, such as curriculum overload, rigid regulatory frameworks, accreditation constraints, and limited teaching hours. At the same time, some interviewees identified facilitators that could enable curricular change, including the use of reaccreditation cycles, incorporation into elective modules, and the role of committed educators or local champions. The theme reflected the tension between preserving formal academic structures and introducing novel, emotionally grounded topics, like second victim experiences. These insights clarified how systemic conditions shaped the capacity for curricular reform and how flexibility and individual advocacy may provide entry points for integration.

#### 3.3.1 Challenges in integrating patient safety topics into existing curricula

Integrating patient safety topics, particularly the second victim phenomenon, into existing curricula presented several challenges. Participants frequently cited the already overloaded curricula and stringent regulatory requirements as significant barriers. In several countries, alignment with the European Union Directive and accreditation demands made even minor curricular changes difficult.

"Well, the curriculum for degree students is insanely full; i.e., in accordance with European Union directives. We already have such strict requirements for everything students need to be taught." (Participant A, Finland)

“Compulsory courses are sometimes more difficult to put into the system… after we build some kind of program, we need to train the faculty… hundreds of instructors that we need to train.” (Participant A, Israel)

“The problem is to introduce it when it is into a compulsory curriculum… either introduce it into some already existing subject and make it part of the subject… The second version would create a compulsory subject, but which would have to take lessons from other subjects, so other subjects would have to give up lessons… which is politically difficult.” (Participant B, Slovakia)

#### 3.3.2 Importance of interdisciplinary collaboration in curriculum development

Participants emphasized the importance of interdisciplinary collaboration in curriculum development to ensure that patient safety education was comprehensive and effective. Interdisciplinary collaboration was seen as a facilitator, while resistance to change from traditional teaching methods was also identified as a barrier. Participants called for innovative and creative approaches to teaching patient safety, moving beyond traditional methods to incorporate more interactive and practical training.

“Students are very grateful when they are faced with examples from practice. They like workshops and way of teaching on some informal (ex-cathedra) way. They like “real life examples” (Participant A, Serbia)

"And we do see differences across specialties. And we also see differences across staff, as they—maybe [pause], maybe the term to use, that becomes a ‘little bit jaded’—are in the career longer, and therefore their awareness decreases.'" (Participant A, Ireland)

“I think it’s always beneficial to have at least an element of interdisciplinary… certain principles… transcend the different professions… So in such cases… it would be very helpful to have a joint element… a blended way… common element…” (Participant A, Malta)

#### 3.3.3 Opportunities for curriculum change: electives, reaccreditation, and faculty initiative

Despite these constraints, participants in several contexts pointed to mechanisms that could facilitate change. Reaccreditation cycles—though infrequent—offered structured windows to propose more substantial curricular revisions. Elective modules were also mentioned as a more flexible avenue for testing or embedding new content, particularly in early implementation phases. Additionally, committed educators, often acting as local champions, were described as instrumental in initiating curriculum reform, especially when working within departmental autonomy or as part of international collaborations.

“We have enough freedom to include the topic during the next accreditation of [the] study program. Change of study program is possible only when we have re- accreditation, every 7 years.” (Participant A, Serbia)

“At least, let's say at the beginning, as an elective subject, it is absolutely possible. And many of those elective subjects that then… grew into compulsory elective, because a lot of students chose them.” (Participant A, Slovakia)

“What we are talking about is to integrate content into existing courses… then it is really our intention to integrate it… the centers of the courses… [and] Dr. John Doe is very much promoting the subject.” (Participant A, Israel)

### 3.4 Theme 3: content and delivery of training

This theme explored participants’ views on the nature and delivery of patient safety and second victim education. Participants emphasized the need for training to go beyond theoretical instruction, advocating for experiential learning, practical coping skills, and emotionally intelligent pedagogy. The theme captured a call for evidence-based, context-sensitive teaching approaches that normalize conversations about error and emotional impact, while fostering professional resilience and safety culture. The focus on pedagogical methods highlighted practical levers and obstacles that educators faced when attempting to implement second victim content meaningfully.

#### 3.4.1 Emphasis on practical coping skills and interdisciplinary training

The content and delivery of patient safety training were highlighted as critical areas needing improvement. Participants stressed the urgent need for practical coping skills and interdisciplinary training, advocating for a shift towards a more hands-on approach in patient safety education. Simulation-based learning, teamwork exercises, and emotionally grounded communication strategies were identified as effective teaching tools.

"The new curriculum will… emphasize this a bit more through simulated case scenarios where people take the parts of the healthcare professions, and they're given instant feedback." (Participant B, Malta)

“I actually created and developed interdisciplinary simulation lessons for the education department for, as it were, nurses in their professional development year, medical candidates, and specialist doctors. Patient safety, teamwork, and communication are key elements of that training.” (Participant A, Iceland)

Maybe a little bit like, you know, empathy… But I need to know how. I have to be able to choose the way. And that's what I think our students don't know and aren't trained [in]…” (Participant B, Slovakia)

#### 3.4.2 Grounding delivery approaches in evolving research culture

In addition to calls for emotionally intelligent and practice-based teaching, several participants emphasized that curricular content on second victim phenomena should be grounded in research. They noted that research not only supports the development of more effective delivery strategies but also lends credibility to the inclusion of such content in formal curricula. In systems where teaching innovation was closely tied to academic evidence, research played a dual role: improving delivery and justifying curricular integration.

"…in the future, more studies related to patient safety will be developed, and when we have that research in particular, as long as these research documents progress, since the teaching is, of course, research-based…"]i.e., research will support and justify the inclusion of patient safety topics in teaching[. (Participant B, Finland)

"I suggested to include that in the curricula, as a separate topic, as well as in the handbook. I have called this topic ‘Professional attention’ [e.g., attention to clinicians' emotional well-being or second victim issues]. Unfortunately, it wasn’t accepted so far. The reason was lack of references." (Participant A, Serbia)

While educators are eager to adopt interactive and emotionally attuned teaching methods, systemic support for research-informed pedagogy may be critical to long-term curricular change.

### 3.5 Theme 4: student and educator engagement and impact

This theme captured participants’ recognition of students as active stakeholders in shaping patient safety education. Educators described how students’ advocacy, expectations, and emotional awareness influenced curricular priorities. While student motivation was seen as a facilitator of change, challenges remained in engaging students meaningfully and aligning content with their developmental readiness. The theme underscored how student-centered approaches, including students’ attitudes, expectations, and emotional awareness, can either reinforce or challenge institutional efforts to embed patient safety and second victim issues into formal education.

#### 3.5.1 Importance of student and educator involvement in curriculum design

Engaging both students and educators in the design and implementation of the curriculum was seen as essential for its success. Participants believed that students would respond positively to innovative and interactive new courses focused on patient safety and the second victim phenomenon. At the same time, they highlighted a gap in educators’ preparedness to teach these topics, suggesting that teacher training and engagement must accompany student involvement. Recognizing and harnessing student motivation were considered critical factors. Participants suggested that actively involving students in curriculum development could help ensure that the educational content is relevant and impactful.

“What is now really happening in this situation, because students really opened up the topic and even students opened up the topic… that teachers should be educated on how to properly teach future doctors.” (Participant A, Slovakia)

“There are the stakeholders, the academic members of staff, but also the students, because the students obviously are an essential part, and they are voice as well…” (Participant A, Malta)

"The students too, who should be involved, and should be an important part in the changes, and our collaboration partners in the practice field." (Participant A, Norway)

#### 3.5.2 Recognition of student motivation and reactions to training

Participants also discussed the potential benefits of providing incentives for student engagement, such as integrating these topics into final thesis projects or offering specialized training modules. Student engagement and motivation were seen as facilitators, as they not only enhanced student involvement but also fostered a deeper understanding and commitment to patient safety.

"…but I am very optimistic because I think the Y generation is much more attentive to this matter of what happens to me in certain situations and how I should deal with them." (Participant B, Israel)

"I have supervised a thesis on this subject. It is not usually done in undergraduate studies…” (Participant A, Spain)

### 3.6 Theme 5: continuous professional development and future directions

This theme addressed the need for ongoing education and systemic reform to ensure healthcare professionals remained equipped to handle patient safety challenges and second victim experiences. Participants stressed the importance of continuous faculty development and institutional support, noting that sustainable change depended on action at both the individual educator level and the broader organizational infrastructure. Lifelong learning, reflective teaching, and a strong institutional culture were all identified as crucial for long-term curricular integration. The findings emphasized that improving how second victim content was taught required investment both in educators’ skills and in the systems that enabled their development.

#### 3.6.1 Recognition of the need for continuous education and updating of healthcare professionals’ competencies

Participants consistently emphasized the need for continuous education and updating of healthcare professionals’ competencies. They highlighted the need for faculty development programs to equip educators with the necessary skills and knowledge to teach patient safety effectively. Ongoing professional development was seen as essential to keep pace with the rapid advancements in healthcare and education. Participants emphasized the need for systematic reforms to ensure that medical education adequately reflected current healthcare challenges. Continuous professional development and systematic reforms were identified as facilitators, preparing future healthcare professionals to prioritize patient safety through continuous learning and adaptation.

"There have to be multiple repeat interventions… if you, for example, deliver training interventions in March, quite a lot of the cohorts that underwent that training has left by July, and therefore you really need to have another intervention in August or September, if you're going to maintain the behaviours that you are hoping for." (Participant A, Ireland)

“Formal education, of course, needs to be constantly improved and expanded according to the needs of society.” (Participant A, Lithuania)

“There is an annual program known in advance, a course for department managers… A course for risk management or patient safety officers in the institution… Workshop for a new employee… There is fine tuning, let's say, for each such target population.” (Participant B, Israel)

“The institution is responsible for supplementary education and certain training for its people, that must be ongoing. And it is, I think, in the simulation centers, where people are being trained in communication and this and that.” (Participant B, Spain)

## 4 Discussion

We have revealed a complex interplay of factors influencing the implementation of patient safety, as well as second victim phenomenon and support training in health professional curricula across European countries. These results both align with and extend previous research in this field, highlighting areas of progress and persistent challenges (Tregunno et al., 2014; Vaismoradi et al., 2020; Carrillo et al., 2022; Gil-Hernández et al., 2024), thus underscoring the importance of addressing this issue from a continental perspective.

Widespread recognition of the impact of adverse events on patients and healthcare workers aligns with the growing body of literature emphasizing the importance of patient safety in healthcare education (Carrillo et al., 2022; Vaismoradi et al., 2020; Jha et al., 2010). However, the limited inclusion of the second victim phenomenon in formal curricula represents a significant gap, echoing findings from previous reserachers who have highlighted the need for more comprehensive patient safety education (Lee and Dahinten, 2023). This gap between awareness and practice highlights the need for structured approaches to incorporating these concepts into health professional education. The European Researchers' Network Working on Second Victims has expanded the second victim concept beyond patient safety incidents to include other highly stressful situations faced by healthcare professionals, where adverse events are one of many contributing factors (Vanhaecht et al., 2022). Preparing future nursing and medical professionals, including residents, to handle these challenges should be an objective that begins in universities and schools, ensuring that they are equipped to manage the emotional and psychological demands of their roles. Additionally, building resilience and providing support to strengthen this capacity when trainees experience serious adverse events during their training is another critical aspect that educational programs should consider (Mira et al., 2024b). Addressing these needs early may better prepare healthcare professionals to manage the pressures and challenges they will inevitably encounter in their careers.

The primary responsibility of healthcare education is to ensure patient safety. Protecting patients, who are the first victims of medical errors, remains paramount. However, the well-being of healthcare providers, who can become second victims, is also critical. If healthcare professionals are not supported following adverse events, their ability to provide safe care may be compromised (Busch et al., 2021; Liukka et al., 2020). The second victim phenomenon highlights the interdependence of patient and provider safety, reinforcing the necessity for educational curricula that address both aspects comprehensively (López-Pineda et al., 2022).

The challenges in integrating patient safety topics into existing curricula, particularly due to overloaded schedules and regulatory requirements, are consistent with findings from previous reserachers (Tregunno et al., 2014). These barriers reflect the broader challenges in medical education reform, as highlighted by researchers such as Lucian Leape and Donald Berwick (Leape and Berwick, 2005). The emphasis on interdisciplinary collaboration in curriculum development aligns with best practices in medical education and could potentially overcome some of these barriers. This approach is supported by studies demonstrating the effectiveness of interprofessional education in enhancing patient safety competencies (Reeves et al., 2016).

The call for more innovative and practical approaches to teaching patient safety reflects a broader trend in medical education towards experiential learning (Balhara et al., 2022; Shin et al., 2021). The focus on interdisciplinary training and practical coping skills could significantly enhance the effectiveness of patient safety education, preparing students for the realities of clinical practice. This approach is supported by researchers who showed the effectiveness of simulation-based training in improving patient safety outcomes (Hegland et al., 2017; McGaghie et al., 2010). Simulation offers a particularly valuable approach, creating a controlled and psychologically-safe environment where students can explore emotionally and ethically complex situations related to adverse events (Catalán et al., 2024). Beyond technical skill development, simulation creates powerful learning opportunities for students to learn about non-technical skills, such as communication, teamwork, leadership, decision-making, and situational awareness. This approach allows students to reflect on clinical decision-making, communication strategies, and emotional reactions in a structured setting that promotes both interprofessional collaboration and psychological resilience. Furthermore, simulation-based education can serve to normalize discussions around emotional responses to adverse events and reduce the stigma that often surrounds healthcare professionals' emotional vulnerabilities (Madsgaard et al., 2022; Oudshoorn et al., 2021). By incorporating these approaches, educational institutions can begin to bridge the gap between theoretical awareness and practical preparedness, potentially mitigating the psychological impact of future adverse events on healthcare professionals.

The recognition of student engagement as a critical factor in curriculum success is noteworthy and aligns with contemporary educational theories emphasizing learner-centered approaches (Docherty et al., 2018). Involving students in curriculum design not only ensures relevance but also fosters a sense of ownership and commitment to patient safety principles. This approach could lead to more effective and sustainable educational interventions, as suggested by researchers on student-centered learning in medical education (Cook-Sather et al., 2014).

The emphasis on continuous professional development and systemic reforms underscores the dynamic nature of healthcare and the need for ongoing adaptation in medical education (Merry et al., 2023). This focus on lifelong learning and systemic change is crucial for creating a culture of safety in healthcare settings, as emphasized by researchers like James Reason (2000) in his work on organizational safety. We suggest that a commitment to lifelong learning and professional development is vital for maintaining high standards of patient safety and care quality, echoing recommendations from bodies such as the World Health Organization and the Institute of Medicine (IOM, 2000; WHO, 2021).

These findings have significant implications for nursing and medical education policy and practice. They suggest a need for a more integrated and comprehensive approach to patient safety education, one that addresses both the technical aspects of safety and the human factors involved, including the second victim phenomenon. This aligns with the growing recognition of the importance of human factors in patient safety (Dekker, 2011; McCulloch, 2024) and recent work on speaking up in healthcare systems (Carrillo et al., 2024; Kane et al., 2023).

The limited inclusion of second victim phenomenon and support training in curricula underscore the need for targeted interventions to address this gap. This aligns with recent initiatives such as the European Researchers' Network Working on Second Victims, which aims to improve awareness and support for second victims across Europe (Mira et al., 2024a). The development of specialized courses and training programs, such as those mentioned in the European Researchers' Network Working on Second Victims initiative, represents a promising step towards addressing this educational need (Guerra-Paiva et al., 2024; Rodriguez and Scott, 2018).

### 4.1 Strengths and limitations

The strengths of this study include its multi-national scope, providing diverse perspectives from 10 European countries, which enhances the generalizability of the findings and offers comprehensive insights into the international landscape of patient safety education. Recruiting healthcare education leaders who were knowledgeable and experienced in medical and nursing education enriched the quality of data collected. The qualitative design with thematic analysis is suitable for exploring scarcely studied issues, and our transparent reporting enhanced the methodological rigor, enabling readers to evaluate the validity and reliability of the findings.

The study also has limitations. Despite purposeful sampling, the sample size of 19 healthcare education leaders may limit the depth and breadth of perspectives represented, potentially overlooking nuances specific to certain regions or institutions. Additionally, while our cross-national design provided valuable comparative insights, we acknowledge the challenge of heterogeneity across the 10 European countries studied. Despite our methodological strategies, variations in healthcare systems, educational frameworks, and cultural approaches to patient safety inevitably influenced participants' perspectives. This heterogeneity may limit the transferability of specific findings to particular national contexts, though the identified common barriers and facilitators likely have broad relevance across European healthcare education settings. As such, the generalizability of our results should be understood as conceptual rather than context-specific, offering a foundation for reflection and adaptation rather than direct replication.

Given that participants were approached by the investigators and selected based on their roles as department heads or senior leaders, there may be a bias towards individuals with certain viewpoints or experiences, potentially influencing the study findings. Conducting interviews in participants' native languages and then translating them to English suggestes the possibility that important local aspects of educational models, university organizational structures, and cultural contexts may have been lost in translation. While we focused on the descriptions of study program leaders, we may have overlooked perspectives from other stakeholders, such as students, frontline healthcare professionals, or patients, whose insights have provided additional depth to the findings. Furthermore, the study was limited to medical and nursing schools, which may not fully capture the broader range of healthcare educational environments or the perspectives of other health professions.

### 4.2 Implications for educational policy

We suggest that while enhancing patient safety remains a primary focus in healthcare education, there is less emphasis on the safety and well-being of healthcare professionals themselves. Recognizing the welfare of healthcare professionals as integral to overall patient care quality is crucial. To address this, interdisciplinary collaboration is needed to incorporate curricular content focused on building resilience in future generations of healthcare professionals. Nursing, in particular, is more exposed to these challenges, which should be highlighted given the demands of the profession. Balancing patient safety with healthcare worker well-being may be essential for creating a sustainable and supportive healthcare environment.

We have highlighted both potentials and challenges associated with curriculum changes. Expanding the content to include the second victim phenomenon is necessary, but the removal of existing content poses a challenge. Strategic decision-making and collaboration among stakeholders are required to navigate these challenges and ensure a comprehensive curriculum.

Advocating for support from influential entities can help overcome resistance to change and facilitate a more inclusive approach. Leaders in policy development, education, and healthcare services play pivotal roles in this effort. Establishing a robust evidence base for integrating stand-alone courses on the second victim phenomenon and patient safety will enhance the credibility and effectiveness of educational initiatives.

## 5 Conclusion

Patient safety education is a critical element in healthcare training, requiring collaborative efforts to integrate principles and practices across diverse programs. Addressing gaps and prioritizing key topics like the second victim phenomenon can better prepare healthcare professionals to deliver safe care. While there is growing recognition of the importance of these topics, there remains a need for comprehensive understanding and formal teaching.

Challenges such as resource constraints, faculty motivation, and overloaded curricula need to be addressed. Leadership in driving curriculum development and interdisciplinary collaboration are crucial. Advocacy for research-based teaching methods and student engagement in curriculum design are essential for effective educational interventions.

Continuous professional development for faculty and ongoing adaptations in healthcare education are necessary to equip future professionals with the skills to prioritize patient safety. We have emphasized the need for curriculum changes and proactive efforts to raise awareness and advocate for patient safety education in healthcare training programs.

## Funding statement

This article is based upon work from COST Action The European Researchers’ Network Working on Second Victims, CA19113, supported by COST (European Cooperation in Science and Technology). http://www.cost.eu

## CRediT authorship contribution statement

**Einav Srulovici:** Writing – original draft, Project administration, Methodology, Data curation, Conceptualization. **Mary-Elizabeth Tumelty:** Writing – original draft, Methodology, Formal analysis, Data curation, Conceptualization. **Ivana Meszaros Skoumalova:** Writing – original draft, Methodology, Formal analysis, Data curation, Conceptualization. **Patricia Vella Bonanno:** Writing – review & editing, Methodology, Data curation, Conceptualization. **Erika Kubilienė:** Writing – review & editing, Methodology, Data curation, Conceptualization. **José Joaquín Mira:** Writing – review & editing, Project administration, Methodology, Data curation, Conceptualization. **Valentina Marinkovic:** Writing – review & editing, Methodology, Data curation, Conceptualization. **Anat Rafaeli:** Writing – review & editing, Methodology, Data curation, Conceptualization. **Reinhard Strametz:** Writing – review & editing, Methodology, Data curation, Conceptualization. **Susanna Tella:** Writing – review & editing, Methodology, Data curation, Conceptualization. **Anu Venesoja:** Writing – review & editing, Methodology, Data curation, Conceptualization. **Žymantė Jankauskienė:** Writing – review & editing, Methodology, Data curation, Conceptualization. **Sandra C. Buttigieg:** Writing – review & editing, Project administration, Methodology, Data curation, Conceptualization.

## Declaration of competing interest

The authors declare the following financial interests/personal relationships which may be considered as potential competing interests:

All authors report financial support was provided by European Cooperation in Science and Technology. If there are other authors, they declare that they have no known competing financial interests or personal relationships that could have appeared to influence the work reported in this paper.
